# Paracrine Effects of FGF23 on the Heart

**DOI:** 10.3389/fendo.2018.00278

**Published:** 2018-05-28

**Authors:** Maren Leifheit-Nestler, Dieter Haffner

**Affiliations:** Department of Pediatric Kidney, Liver and Metabolic Diseases, Pediatric Research Center, Hannover Medical School, Hannover, Germany

**Keywords:** fibroblast growth factor 23, cardiac remodeling, left ventricular hypertrophy, cardiac fibrosis, endothelial dysfunction, autocrine, paracrine

## Abstract

Fibroblast growth factor (FGF) 23 is a phosphaturic hormone primarily secreted by osteocytes to maintain phosphate and mineral homeostasis. In patients with and without chronic kidney disease, enhanced circulating FGF23 levels associate with pathologic cardiac remodeling, i.e., left ventricular hypertrophy (LVH) and myocardial fibrosis and increased cardiovascular mortality. Experimental studies demonstrate that FGF23 promotes hypertrophic growth of cardiac myocytes *via* FGF receptor 4-dependent activation of phospholipase Cγ/calcineurin/nuclear factor of activated T cell signaling independent of its co-receptor klotho. Recent studies indicate that FGF23 is also expressed in the heart, and markedly enhanced in various clinical and experimental settings of cardiac remodeling and heart failure independent of preserved or reduced renal function. On a cellular level, FGF23 is expressed in cardiac myocytes and in other non-cardiac myocytes, including cardiac fibroblasts, vascular smooth muscle and endothelial cells in coronary arteries, and in inflammatory macrophages. Current data suggest that secreted by cardiac myocytes, FGF23 can stimulate pro-fibrotic factors in myocytes to induce fibrosis-related pathways in fibroblasts and consequently cardiac fibrosis in a paracrine manner. While acting on cardiac myocytes, FGF23 directly induces pro-hypertrophic genes and promotes the progression of LVH in an autocrine and paracrine fashion. Thus, enhanced FGF23 may promote cardiac injury in various clinical settings not only by endocrine but also *via* paracrine/autocrine mechanisms. In this review, we discuss recent clinical and experimental data regarding molecular mechanisms of FGF23’s paracrine action on the heart with respect to pathological cardiac remodeling.

## Fibroblast Growth Factors (FGFs) and Their Receptors

The FGF family consists of 22 members classified into seven subfamilies due to their phylogenetic relationship (1). Based on their mechanism of function, FGFs are further divided into paracrine, endocrine, and intracrine FGFs (Table 1) (2–5). Paracrine FGFs exhibit a heparan sulfate-binding site at their C-terminal region and bind to FGF receptors (FGFR) on the cell surface using heparan sulfate as a co-factor to mediate local biological activities (6). In contrast to paracrine FGFs, endocrine FGFs display a klotho-binding site at their C-termini, and due to their very low heparan sulfate-binding affinity, endocrine FGFs are directly secreted into the blood and exert their functions as endocrine hormones in various target tissues using α-klotho or β-klotho as co-receptors for FGFRs (7–10). Intracrine FGFs are not secreted and exert their biological functions within the same cell (11). While not all FGFs are endogenously expressed in the heart of humans and rodents (12), it was shown that FGF2, FGF3, FGF8, FGF9, FGF10, FGF16, FGF15/19, FGF21, and FGF23 all act on the heart in a paracrine or endocrine manner to induce physiological or pathological pathways in heart development, health, and disease (Table 1) (5, 13). The specific role of these FGFs and their respective FGFRs in the heart are nicely discussed in more detail in two reviews by Itoh and colleagues (5, 13).

**Table 1:** Classification of fibroblast growth factor (FGF) family members according to their mechanistic function.

Function	FGF
Paracrine	FGF1, **FGF2**, **FGF3**, FGF4, FGF5, FGF6, FGF7, **FGF8**, **FGF9**, **FGF10**, **FGF16**, FGF17, FGF18, FGF20, FGF22
Endocrine	**FGF15/19**, **FGF21**, **FGF23**
Intracrine	FGF11, FGF12, FGF13, FGF14

*Bold printed FGFs play a role in heart development, health, or disease conditions*.

In mammals, due to alternative splicing, seven FGFRs are generated from four different FGFR genes (*FGFR1, FGFR2, FGFR3, FGFR4*) with different FGF-binding affinity (14). Paracrine FGFs bind heparan sulfate to promote an FGF/FGFR/heparan sulfate complex while endocrine FGFs bind α-klotho or β-klotho to induce a complex consisting of FGF/FGFR/klotho, which both directly phosphorylate the FGFR intracellular tyrosine kinase domain. After FGFR activation, FGFR substrate 2α (FRS2α) is activated downstream, thereby inducing rat sarcoma protein (RAS)-mitogen-activated protein kinase (MAPK), phosphoinositide 3-kinase (PI3K)-AKT serine/threonine kinase (AKT), or signal transducer and activator of transcription (STAT) signaling within the cell, respectively (3, 4, 15, 16). In addition, phospholipase Cγ (PLCγ) can also be phosphorylated due to FGFR activation leading to activation of calcium-dependent signaling mediating cell motility (15). In the cardiovascular system, FGFR1c, FGFR2b, FGFR2c, FGFR3c, and β-klotho are physiologically expressed at very high levels while FGFR1b, FGFR3b, FGFR4, and α-klotho are only moderately expressed (12).

## Fibroblast Growth Factor 23

FGF23 belongs to the endocrine FGF family and mainly functions as a hormone-like FGF. It is mainly expressed and secreted by osteocytes in the bone as a 32-kDa phosphaturic and calcitriol inhibitory protein regulating phosphate homeostasis (17). FGF23 exists in a full-length biologically active form and can be cleaved into N- and C-terminal fragments. The classic target organs of FGF23 are the kidney and the parathyroid glands where it exerts its physiological function through FGFRs with α-klotho as a co-factor (9, 18). The FGFR-binding site is located in the N-terminus while the α-klotho binding site is located in the C-terminus. The proprotein convertase cleavage site is stabilized by O-linked glycosylation *via* Galnt3 (19). FGF23 acts on the kidney *via* FGFR1c/α-klotho/MAPK signaling to regulate phosphate and vitamin D metabolism. It reduces renal phosphate reabsorption by suppression of sodium phosphate co-transporters NaPi-2a and NaPi-2c, thereby lowering serum phosphate levels (17, 20). Moreover, FGF23 decreases active vitamin D synthesis by downregulation of 1α-hypdroxylase and upregulation of 24-hydroxylase resulting in low levels of 1,25(OH)_2_D_3_ in serum (17). In the parathyroid gland, FGF23 inhibits the secretion of parathyroid hormone (21).

FGF23 expression is almost absent in the central nervous system, endocrine non-reproductive system, and metabolic system, and only minimally expressed in the gastrointestinal system, immune system, reproductive system, and cardiovascular system in healthy adults (12). In pathological conditions, FGF23 was shown to be excessively enhanced in bone (22), heart (23–26), liver (27), and kidneys (28, 29–32).

## FGF23 and the Cardiovascular System

Pathological cardiac remodeling, i.e., left ventricular hypertrophy (LVH), myocardial fibrosis, and vascular calcification are the major cardiovascular pathologies in the general population and in patients with chronic kidney disease (CKD) presenting with 15–21% and up to 90% LVH, respectively (33). Since α-klotho as a specific co-receptor for FGF23 is not expressed in human and rodent hearts (12, 25, 34), direct effects of endocrine-acting FGF23 on the cardiovascular system were not supposed for a long time. However, the causes of cardiovascular disease pathologies are multifactorial. In 2008, FGF23 was discussed for the first time as a new mediator for the progression of LVH in CKD (35). In recent years, recent clinical and experimental studies demonstrated positive associations between endocrine-acting FGF23, cardiac remodeling, and endothelial dysfunction in pathological conditions in humans and rodents. In 2011, Faul and colleagues presented for the first time that administration of FGF23 directly induces hypertrophic growth of isolated neonatal rat cardiomyocytes *in vitro* and LVH *in vivo* in an FGFR-dependent but α-klotho-independent manner (34). In contrast to the established FGF23/FGFR/α-klotho signaling complex primarily mediating the induction of RAS/MAPK pathway (9), FGF23/FGFR activates PLCγ in cardiac myocytes and induces hypertrophic cell growth using calcineurin/nuclear factor of activated T cell (NFAT) signaling in the absence of α-klotho (34). Interestingly, these effects were independent of blood pressure levels. Furthermore, it was shown that FGF23 increases intracellular calcium levels in cardiac myocytes *in vitro* and promotes contractility of murine cardiac myocytes and ventricular muscle strips *ex vivo* α-klotho independently (36). Thus, the α-klotho-independent action of FGF23 on the heart became more and more likely. In 2015, Grabner et al. identified the FGFR4 isoform as specific FGFR in the heart mediating FGF23’s pro-hypertrophic action without α-klotho (37). In recent years, the importance of the role of enhanced *circulating* FGF23 on the development of cardiac remodeling was thoroughly demonstrated in patients with CKD (34, 38–43) and end-stage renal disease (ESRD) (34, 35, 44). Reaching serum levels of up to 1,000-fold higher than in healthy individuals, FGF23, and associated alterations in mineral metabolism, including hyperphosphatemia, hypercalcemia, secondary hyperparathyroidism, vitamin D, and klotho deficiency, are associated with uremic cardiomyopathy, LVH, premature death, and all cause mortality in CKD (35, 38, 41, 45–48).

Clinical studies further indicate that circulating levels of FGF23 are also elevated in patients with dilated cardiomyopathy, ischemic heart disease, acute decompensated and chronic heart failure (HF), atrial fibrillation, and cardiogenic shock, despite normal renal function, suggesting that enhanced cardiac synthesis of FGF23 may cause elevated circulating FGF23 levels and thereby promote cardiac injury (49–61). The endocrine action of FGF23 on the heart and other organ systems was previously described in detail (62–69).

## Paracrine Action of FGF23 on the Heart

It was suggested that FGF23 is not just a biomarker of increased risk but also a direct inducer of cardiac injury in the settings of CKD or HF (34, 70–72). Although FGF23 was discovered in 2001 as an endocrine hormone (73), cardiac FGF23 may be upregulated in certain clinical settings and promote cardiac remodeling *via* autocrine and/or paracrine mechanisms.

### Cardiac FGF23 and LVH

Recently, we analyzed myocardial autopsy samples of deceased patients with childhood-onset ESRD and investigated the well-established FGF23/FGFR4 signaling cascade mediating cardiac hypertrophy. We demonstrated that FGF23 is expressed by cardiac myocytes in humans as the full-length biologically active protein (25). Moreover, cardiac FGF23 and FGFR4 are strongly enhanced in heart tissue of CKD patients when compared to age- and sex-matched controls and both positively associate with the development of LVH. As expected, klotho is not expressed in the human heart on mRNA level. However, soluble klotho protein can be clearly detected in myocardial tissue of CKD patients by Western blot and immunohistochemical analysis. The amount of soluble klotho in myocardial tissue is found to be reduced in CKD patients, and negatively associated with the duration of ESRD, dialysis vintage, and cardiac myocyte cross-sectional area. Interestingly, cardiac expression of FGF23, FGFR4, and the amount of soluble klotho in cardiac tissue are found to be dysregulated in dialysis patients and ameliorated in patients with functioning renal allografts. Similarly, FGF23/FGFR4-associated calcineurin/NFAT pathway and the expression of pro-hypertrophic markers are normalized after kidney transplantation. Multiple linear regression analysis points out that cardiac FGF23 expression and the amount of soluble klotho protein in cardiac tissue are independent predictors of cardiac myocyte cross-sectional area while the mode of renal replacement therapy (RRT) at time of death is found to be the only independent predictor for the induction of cardiac FGF23 synthesis. The amount of soluble klotho protein in the cardiac tissue of CKD patients correlates with the duration of ESRD and the mode of RRT at time of death, and finally, cardiac FGFR4 expression correlates with estimated glomerular filtration rate, mode of RRT at time of death, cumulative time spent on dialysis, and cardiac FGF23 expression levels. The above-mentioned findings are supported by a recent study investigating myocardial tissue of 5/6 nephrectomized (Nx) rats, which is a well-established model of experimental uremia (26). Indeed, cardiac *Fgf23* gene expression is up-regulated in myocardial tissue of 5/6Nx rats compared with sham operated animals. Furthermore, experimental uremia induces full-length cardiac Fgf23 protein demonstrated by Western blot, which positively correlates with the heart weight to body weight ratio, and enhanced β*MHC* and *BNP* expression levels. Although these association studies do not prove causality, data suggests the existence of a *paracrine* FGF23 mechanism in the heart mediating LVH in uremic cardiomyopathy.

Studies in patients with acute decompensated heart failure (ADHF) point out that circulating FGF23 levels are significantly enhanced in ADHF compared to controls, whereas myocardial *FGF23* gene expression is not consistently altered (60). However, the investigated study cohort is rather small and *FGF23* mRNA levels greatly vary within both groups, which may have biased the results of this study. Since ADHF is defined as sudden or gradual onset of HF symptoms (74), the degree of HF progression may also define the strength of cardiac FGF23 induction. Nevertheless, FGF23 is detected in myocardium of both HF and normal patients by quantitative real-time PCR confirming the presence of FGF23 in the human myocardium. In addition, immunohistochemical analysis detects FGF23 expression in the cardiac myocytes of normal myocardial tissue and in failing hearts.

A recent study by Slavic and colleagues investigated cardiac and circulating FGF23 in experimental heart hypertrophy (75). They showed that increased afterload due to transverse aortic constriction (TAC) in mice induces an increase in circulating intact Fgf23 serum levels within 24 h after TAC, which maintains for at least 4 weeks post-surgery. TAC significantly increases cardiac *Fgf23* gene transcripts while bone *Fgf23* production is higher overall compared to the heart but not significantly stimulated by TAC. Furthermore, pressure overload dramatically reduces the cleavage of Fgf23 in serum. Interestingly, expression of *Galnt3*, which O-glycosylates and thereby stabilizes Fgf23 protein, is increased in the heart post-TAC but unchanged in bone. By contrast, expression of *Fam20c* and *Furin*, promoting Fgf23 cleavage, remain unchanged in heart tissue of mice after TAC. Taken together, these data suggest that cardiac FGF23 synthesis is induced due to pressure overload and reduced FGF23 cleavage further contributes to enhanced intact FGF23 in the heart. Importantly, the up-regulation of cardiac and serum intact Fgf23 is not due to alteration of mineral metabolism as serum levels of phosphate, calcium, sodium, potassium, and iron remain unchanged in mice after TAC compared to sham-operated animals. Instead, serum aldosterone levels, shown to stimulate FGF23 expression *in vitro* (76), are induced in TAC-operated mice. To confirm whether induction of renin–angiotensin–aldosterone system (RAAS) triggers pressure overload-induced up-regulation of cardiac Fgf23, the authors treated wild-type mice with spironolactone for 2 weeks post-TAC surgery (75). Administration of spironolactone inhibits the induction of circulating intact Fgf23 levels in TAC mice and reduces the mRNA expression of *Fgf23* in bone but not in the heart, suggesting that a pressure overload-induced increase in bone *Fgf23* synthesis is driven by aldosterone secretion in TAC mice. Interestingly, heart weight to body weight ratio, cardiac myocyte size, end-diastolic left ventricular wall thickness, fractional shortening, and mean blood pressure after TAC are not improved by aldosterone inhibition, indicating that increased systemic Fgf23 may be less important in the development of pressure overload-induced cardiac hypertrophy. However, TAC-induced expression of cardiac *Fgfr1, Fgfr3*, and *Fgfr4*, nuclear localization of NFAT protein within the cardiac myocytes, *Rcan1* induction, and consequently cardiac hypertrophy and fibrosis are also present in global homozygous *Fgf23^−/−^*/vitamin D receptor (VDR)^Δ/Δ^ or *Klotho^−/−^*/VDR^Δ/Δ^ double knockout mice compared to respective wild-type mice. Neither deletion of Fgf23 protected against pressure overload-induced cardiac hypertrophy and fibrosis, nor klotho deficiency exacerbated TAC-induced pathological hypertrophic phenotype in mice. Thus, FGF23 would appear to be a consequence of pressure overload-induced hypertrophy and perhaps not the initial inducer, suggesting that additional factors could mediate hypertrophic responses during pressure overload independent of FGF23.

FGF2 is expressed in cardiac myocytes and fibroblasts, and angiotensin II or adrenergic stimulation induces FGF2 synthesis in cardiac myocytes, which in turn stimulates itself in a feed-forward loop (77). High blood pressure induces LVH *via* up-regulation of FGF2, which is shown to be necessary for hypertrophic development in the TAC model (78). Neonatal and adult cardiac fibroblasts mainly express the high-molecular weight isoform of FGF2, which is stimulated and secreted through angiotensin II treatment, thereby acting on cardiac myocytes *via* induction of a fetal gene program resulting in cardiac hypertrophy (79). However, neither spironolactone treatment nor FGF23 knockout alters the induction of FGF2 in the high blood pressure-induced cardiac hypertrophy through TAC (75). Interestingly, it was shown that high-molecular weight FGF2 isoform directly stimulates FGF23 synthesis in the bone (80, 81). Whether up-regulation of FGF2 in the heart due to pressure overload-induced cardiac hypertrophy after TAC is also responsible for the induction of cardiac FGF23, and if so, the role of cardiac FGF23 in this setting has to be further elucidated.

However, these conclusive findings coincide with clinical and experimental studies showing that enhanced FGF23 serum levels and/or the induction of cardiac FGF23 synthesis are not associated with high blood pressure-induced LVH (25, 34, 37). In addition, the fact that cardiac FGF23 is still enhanced after mineralocorticoid receptor (MR) blockade while bone FGF23 and systemic FGF23 are reduced supports our hypothesis that induction of cardiac FGF23 may have additional blood pressure-independent paracrine effects on the heart, which needs to be addressed in future studies.

### Cardiac FGF23 and Myocardial Fibrosis

The role of circulating and/or cardiac FGF23 in the progression of cardiac fibrosis and its potential effects on myocardial fibroblasts promoting cardiac diastolic function is largely unknown. Initial experimental data in rodents demonstrated that FGF23 stimulates the renal expression and production of transforming growth factor β (TGF-β) (82, 83). TGF-β is a classic cytokine involved in different signaling pathways, which is shown to induce fibrosis in various tissues.

Recently, Smith et al. reported that FGF23 is synthesized locally by renal tubules and activates injury-primed fibroblasts (31). Moreover, FGF23 stimulates gene expressions involved in TGF-β signaling pathways *via* FGFR4-dependent activation of NFAT and enhancement of TGF-β autoinduction in injury-primed renal fibroblasts (84). These data suggest a paracrine mechanism of FGF23 in the kidney acting on renal fibroblasts to induce pro-fibrotic signaling pathways.

In the heart, cardiac FGF23 expression is upregulated in post-myocardial infarction (MI) in rodents and mediates cardiac fibrosis through the activation of β-catenin (24). β-catenin is a pro-fibrotic factor cross talking with TGF-β signaling (83, 85, 86). Hao and colleagues demonstrated that FGF23 is endogenously expressed in neonatal rat cardiac myocytes and fibroblasts, adult mouse cardiac fibroblasts (AMCF), and the adult mouse heart (24). They also confirm the findings of Slavic et al. mentioned above (75), showing an increase in cardiac FGF23 mRNA and protein levels in pressure overload-induced failing mouse hearts after TAC. In addition, FGF23 plasma levels are significantly induced post-MI compared to sham-operated mice. Angiotensin II and phenylephrine, both pro-fibrotic and pro-hypertrophic stimulators, clearly up-regulate *FGF23* expression in AMCF. Furthermore, stimulation with FGF23 promotes proliferation, collagen I and III synthesis, and β-catenin activation in AMCF. Cardiac-specific overexpression of FGF23 using adeno-associated virus carrying FGF23 (AAV-FGF23) in mice followed by MI or ischemia reperfusion significantly enhances the activation of cardiac β-catenin, TGF-β, collagen I and III, and cardiac fibrosis in mice injected with AAV-FGF23 compared to negative controls. It is important to note that the injection of AAV-FGF23 does not result in enhanced β-catenin, TGF-β, or collagen I and III expression levels in sham-operated mice compared with respective control animals. Thus, endogenous cardiac FGF23 synthesis may only impair myocardial fibrosis after MI or ischemia reperfusion through induction of paracrine signaling pathways, including activation of β-catenin and TGF-β. However, the activation of β-catenin-mediated induction of TGF-β and collagen synthesis by FGF23 under healthy conditions remains controversial.

Andrukhova and colleagues demonstrated that both skeletal and cardiac Fgf23 expression are increased in mice and rats after MI independent of changes in serum soluble klotho levels (23). In addition, they found increasing serum intact Fgf23 levels in mice and rats after experimental MI, and immunohistochemical staining of heart tissue clearly reveals expression of Fgf23 in left ventricular cardiac myocytes after MI in mice and rats but not in infiltrating leukocytes within the infarct scar, suggesting that cardiac myocytes are the major cellular source of increased cardiac Fgf23 expression post-MI. Importantly, despite the induction of Fgf23, serum levels of PTH, phosphate, calcium, and sodium, and urinary excretion of phosphate, calcium, and sodium remain unchanged post-MI. In addition, biochemical markers of bone metabolism, including urinary excretion of collagen cross-link deoxypyridinoline, serum alkaline phosphatase, Dickkopf-1, and osteoprotegerin, do not differ between mouse and rats post-MI compared with sham control-operated animals. Although the molecular link between cardiac injury and enhanced Fgf23 levels remains to be further elucidated, these data strongly support the hypothesis that cardiac FGF23 expression is linked to pathological cardiac changes and may exert paracrine mechanisms in the heart.

In an extension to experimental studies in rats and mice, we recently analyzed the myocardial tissue of CKD patients and investigated cardiac FGF23 expression and myocardial fibrosis (87). Cardiac FGF23 synthesis and accumulation of fibrillar collagen fibers are strongly enhanced in CKD patients on dialysis treatment compared to controls. Cardiac fibrosis correlates with duration of dialysis and soluble klotho deficiency detected by immunohistochemical staining of klotho protein in heart tissue sections. Interestingly, no significant correlations between cardiac *FGF23* expression and fibrosis could be detected in CKD patients. By performing human fibrosis RT profiler PCR array analyses of myocardial tissue in these patients, angiotensinogen (*AGT*), and angiotensin II-driven pathways known to promote fibrotic response, including collagen remodeling, activation of TGF-β/TGF-β receptor/Smad complexes, and induction of pro-hypertrophic and pro-fibrotic growth factors endothelin-1 and connective tissue growth factor (*CTGF*), are significantly enhanced in patients on dialysis treatment. Although cardiac fibrosis positively correlates with the induction of *AGT* in the heart, soluble klotho deficiency but not endogenous FGF23 expression is shown to be correlated with enhanced cardiac TGF-β/TGF-β receptor pathway. We performed further *in vitro* studies using neonatal rat ventricular myocytes (NRVM) and fibroblasts (NRCF) in order to discriminate which cell type express FGF23 in the heart and may induce pro-fibrotic signaling. Hereby, we could clearly detect endogenous *Fgf23* mRNA expression in NRVM but not in NRCF, which is in contrast to Hao et al. also demonstrating FGF23 production in cardiac fibroblasts of neonatal rats and adult mice (24). However, treatment of NRCF with recombinant FGF23 significantly stimulates collagen remodeling, promotes the induction of pro-fibrotic TGF-β/TGF-β receptor/Smad complexes, and induces *Ctgf* and *endothelin-1* expression *in vitro* (87). Importantly and in line with the Hao et al. study mentioned above, FGF23 significantly stimulates proliferation and migration of NRCF. In addition, FGF23 induces angiotensinogen expression in cardiac myocytes, promotes extracellular matrix remodeling, and induces pro-fibrotic and pro-hypertrophic growth factors in NRVM while TGF-β/TGF-β receptor signaling unchanged. Taken together, our data suggest that secreted by cardiac myocytes, FGF23 may directly target cardiac fibroblasts and promote pro-fibrotic mechanisms in a paracrine manner. This mechanism in concert with its autocrine induction of fibrotic and hypertrophic-related factors in cardiac myocytes, cardiac FGF23 may act on both cardiac cell types and thereby promote cardiac hypertrophy and myocardial fibrosis in a paracrine/autocrine fashion.

### Cardiac FGF23 and Endothelial Dysfunction

Clinical and experimental studies demonstrated that high FGF23 serum levels, hyperphosphatemia, and klotho deficiency are strongly associated with cardiovascular complications in CKD patients (71). Elevated FGF23 serum levels associate with endothelial dysfunction and small vessel disease, stroke, and brain infarction in patients with CKD stage 3–4 (40) and in the general population (58, 61, 88), respectively. Whether enhanced circulating levels of FGF23 also impact on vascular calcification is still an open question (39, 42). Voigt and colleagues identified FGF23 accumulation in calcified carotid atherosclerotic lesions from subjects with normal renal function (89). In addition, a recently published study by van Venrooij et al. evaluated the relationship between kidney function, vascular calcification, and FGF23 expression in patients with heart disease (90). They detected FGF23 protein expression by immunohistochemistry in coronary arteries of patients undergoing heart transplantation, which associates with declining renal function. Vascular FGF23 expression and FGFR1, FGFR3, and α-klotho are observed in both tunica intima and tunica media in areas of calcification. Interestingly, co-immunostaining for FGF23 and CD68 demonstrates co-localization of FGF23 with inflammatory macrophages. Quantitative real-time PCR analysis confirms the gene expression of FGF23 in human coronary artery tissue, also suggesting a potential paracrine effect of FGF23 in vessels.

We performed *in vitro* studies using human coronary artery endothelial cells (HCAEC) in order to investigate the underlying molecular pathways of FGF23-mediated endothelial cell function in the presence and absence of its co-receptor α-klotho (91). First, we confirmed the previously reported findings (90) that FGFR1 and α-klotho are present in the intima of human coronary arteries. In addition, cultured HCAECs express FGFRs and α-klotho, and FGF23 specifically stimulates phosphorylation of FGFR1 and thereby activates the receptor, suggesting that vascular endothelial cells are a target for paracrine FGF23 signaling. Although α-klotho is not altered by FGF23 treatment on mRNA or protein level, FGF23 increases the expression of ADAM17, responsible for cleavage of membrane bound α-klotho, and consequently the secretion of soluble α-klotho. Investigating endothelial cell function targeted by FGF23, we showed that FGF23 activates intracellular signaling to induce the release of nitric oxide (NO) *via* activation of AKT/endothelial NO synthase (eNOS) pathway *in vitro* in the presence of α-klotho. Furthermore, FGF23 treatment enhances the formation of reactive oxygen species (ROS) through induction of NADPH oxidase 2 (Nox2)/p67phox/p47phox/Rac1 signaling complex. In addition, FGF23 stimulates the expression of ROS detoxifying enzymes manganese superoxide dismutase and catalase by activating Foxo forkhead transcription factor 3a (Foxo3a) in the presence of α-klotho, indicating that FGF23-induced ROS production is being neutralized by antioxidant mechanisms. Co-treatment with FGF23 and pan-FGFR inhibitor blunts all NO synthesis, ROS formation, and ROS degradation. Taken together, these data suggest that FGF23 effects on HCAEC mediating endothelial function in the presence of α-klotho are FGFR dependent. Inhibition of α-klotho using a neutralizing antibody specifically blocks FGF23-mediated activation of AKT/eNOS and consequently the release of NO. Most importantly, blocking of α-klotho further inhibits the expression of SOD2 and catalase induced by FGF23 but does not affect FGF23 stimulated Nox2 signaling and ROS formation. Our data suggest that in states of Klotho deficiency, e.g., CKD, FGF23-induced NO synthesis is blunted and ROS formation overrules ROS degradation. Consequently, enhanced circulating as well as locally synthesized FGF23 may promote endothelial dysfunction and further impact on the progression of cardiovascular disease in CKD. However, the hypothesized paracrine signaling mechanism of FGF23 mediating functional response in vessels has to be further proven in experimental animal models.

### FGF23 and Chronic Inflammation

Cardiac hypertrophy and myocardial fibrosis can be promoted by pro-inflammatory cytokines, such as TNF-α or IL-6, and pro-fibrotic molecules including TGF-β and angiotensin II (92). Inflammatory markers, including IL-6, CRP, TNF-α, and fibrinogen, are independently associated with circulating levels of FGF23 in patients with CKD, and FGF23 associates with a greater odds ratio of severe inflammation in these patients (93). Furthermore, FGF23 directly stimulates hepatic secretion of pro-inflammatory cytokines in wild-type mice and in cultured hepatocytes by activating FGFR4 independent of α-klotho (94), indicating a novel mechanism of FGF23 and chronic inflammation. Furthermore, chronic inflammation in cardiac fibroblasts was shown to induce the expression of FGF23 in the heart (95), suggesting a paracrine signaling mechanism of chronic inflammation inducing cardiac FGF23. However, hard evidence from experimental studies is lacking.

The cytokine oncostatin M (OSM) belongs to the IL-6 family and is involved in various biological processes, including inflammation and ischemic heart disease (96). OSM is further shown to be a major mediator of cardiac remodeling (97). After binding to a receptor heterodimer consisting of gp130 and OSM receptor β, OSM induces a Janus Kinase/STAT, MAPK, or PI3K/AKT signaling pathway, respectively (98–100), also all known to be induced by FGF/FGFR complexes. Richter and colleagues find that FGF23 is present in cardiac myocytes of patients with ischemic cardiomyopathy, myocarditis, and dilated cardiomyopathy (101). In addition, adult rat cardiac myocytes clearly expresses *Fgf23* on the mRNA level, which is excessively induced by OSM treatment *in vitro* (102). Moreover, cultured OSM-treated non-cardiac myocytes, mainly fibroblasts, also synthesize FGF23. However, FGF23-expressing non-cardiac myocytes are only rarely observed in diseased human heart tissue (101). ELISA quantifications of cardiac myocyte cell supernatant demonstrates significantly increased C-term FGF23 concentrations due to OSM, and Western blot analysis revealed that OSM stimulates both unmodified full-length FGF23 protein and cleaved FGF23 fragments in cell lysates of adult rat cardiac myocytes (102). Similarly, in corresponding cell supernatant C- and N-terminal FGF23 fragments are clearly detected. Inflammatory HF in mice caused by macrophages infiltration of the myocardium shows a strong increase in FGF23 expression and secretion in cardiac myocytes, which is associated with macrophage infiltration and activation of OSM receptor β cascade leading to cardiac myocyte remodeling with enhanced α-smooth muscle actinin expression, STAT3 phosphorylation, induction of atrial natriuretic peptide (ANP), and destrin. These findings suggest that FGF23 is endogenously expressed in cardiac myocytes in inflammatory HF of humans and rodents and associates with macrophage infiltration and OSM receptor activation.

FGF23 is also shown to be expressed in pro-inflammatory M1 macrophages and specifically up-regulated by lipopolysaccharide and interferon gamma (IFN-γ) *via* NF-κB and JAK/STAT1 pathways, respectively (103), indicating that FGF23 produced by M1 macrophages may modulate pro-inflammatory functions *in vitro* and may support innate immune response to tissue injury. Therefore, it is possible that endogenous expression and secretion of FGF23 by infiltrating macrophages induce cardiovascular and kidney disease progression through induction of inflammation and fibrosis.

### Cardiac FGF23 and RAAS

The activation of RAAS was shown to have direct hypertensive effects and stimulates pro-inflammatory, pro-hypertrophic, and pro-fibrotic pathways in cardiac cells, which are pharmacologically suppressed by angiotensin-converting enzyme (ACE) inhibitors, angiotensin receptor blockers (ARB), and MR antagonists (92). The initial RAAS factor is the induction of angiotensinogen expression. Renin converts angiotensinogen into angiotensin I, and angiotensin I is further metabolized to angiotensin II by ACE, finally affecting myocardial hypertrophy and fibrosis. In contrast, angiotensin-converting enzyme 2 (ACE2) degrades angiotensin I and II into angiotensin 1–9 and 1–7, respectively, acting as vasodilatory and hypotensive reagents. It was shown that FGF23 directly stimulates RAAS through inhibition of ACE2 (82) and recent studies indicate that activation of RAAS in turn can also induce FGF23 synthesis (104). In addition, renin is suppressed by 1,25(OH_2_)D_3_ thereby avoiding RAAS activation (66). Since FGF23 suppresses 1,25(OH_2_)D_3_ metabolism in the kidney, one may speculate that FGF23 also alters RAAS indirectly through inhibition of 1,25(OH_2_)D_3_. However, the precise mechanisms of how cardiac FGF23 interacts with the local RAAS in the heart and thereby induce cardiac remodeling and impair diastolic function, remains to be further elucidated.

**Figure 1:**
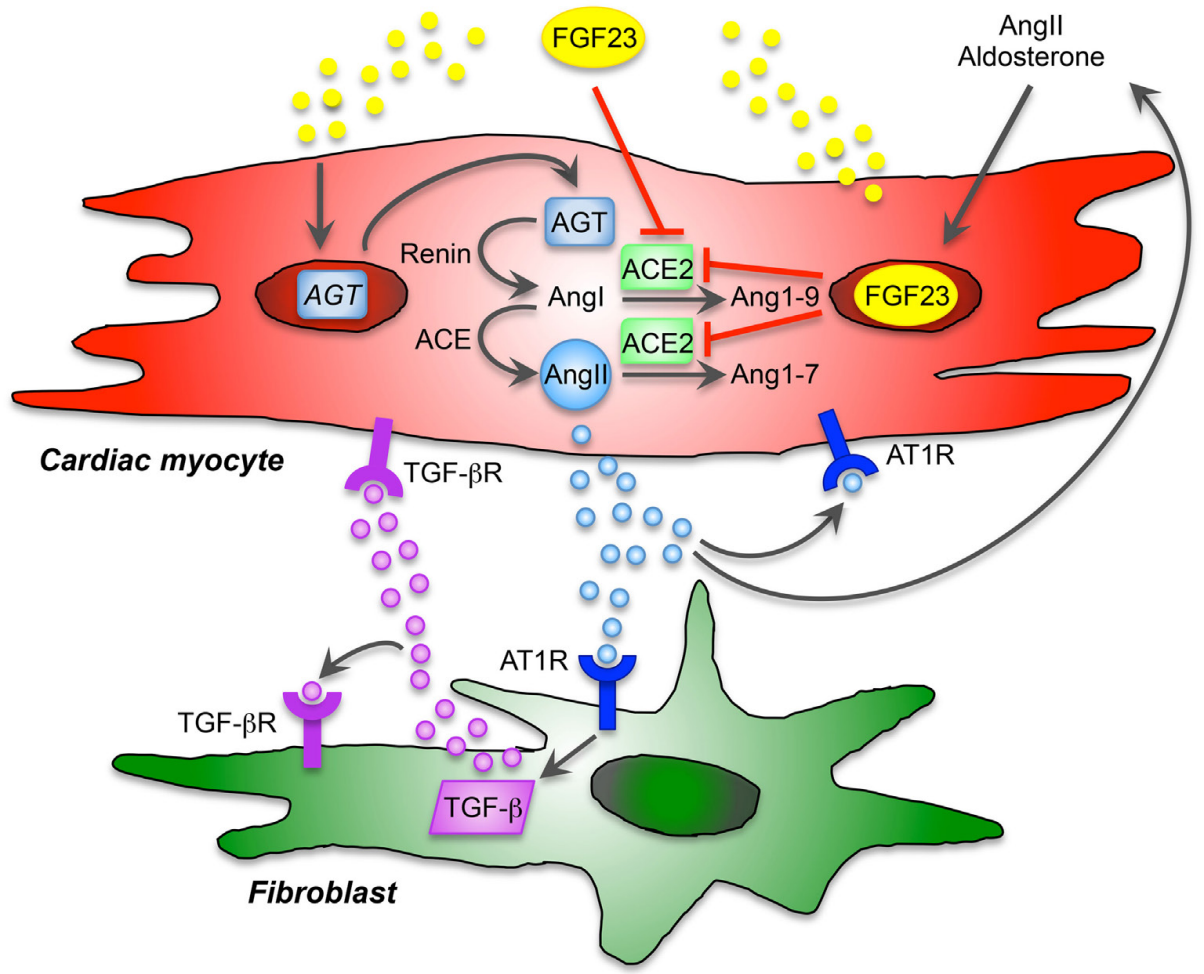
Proposed model of cardiac FGF23 and local renin-angiotensin-aldosterone system (RAAS) in the heart. The local, non-canonical activation of RAAS directly contributes to cardiac remodeling and hypertrophy and affects different cardiac cell types. Angiotensin II (AngII) type 1 receptor (AT1R) is expressed by cardiac myocytes and fibroblasts and thereby displays an important role in maladaptive remodeling. AngII and aldosterone are stimulators of FGF23 expression and secretion, and cardiac FGF23 directly induces the expression of angiotensinogen (*AGT*) mRNA in cardiac myocytes. After activation, AGT is converted into AngI by renin and further to AngII through angiotensin-converting enzyme (ACE). Angiotensin-converting enzyme 2 (ACE2), which physiologically cleaves AngI and AngII counteracting the pathological effects of AngII, is inhibited by FGF23. Thus, FGF23 directly contributes to RAAS activation *via* induction of AGT and suppression of ACE2 in cardiac myocytes resulting in enhanced AngII and aldosterone synthesis. AngII binds to AT1R and promotes hypertrophic response in cardiac myocytes in an autocrine/paracrine manner and stimulates cardiac FGF23 in a feed-forward loop. In addition, FGF23-mediated activation of RAAS and consequently increased AngII contribute to cardiac fibrosis *via* binding of AngII to AT1R on cardiac fibroblasts, which directly promotes differentiation and matrix production in cardiac fibroblasts. In addition, AngII/AT1R induces the expression of transforming growth factor β (TGF-β), which in turns binds to TGF-β receptor (TGF-βR) on cardiac fibroblasts to induce itself or stimulate hypertrophic molecules and signaling in cardiac myocytes. Taken together, endogenous expression of FGF23 in cardiac myocytes is directly involved in the activation of local RAAS contributing in pathological hypertrophy and fibrosis in a paracrine manner.

Angiotensin II and aldosterone, as active RAAS components, promote vascular and myocardial fibrosis as well as cardiac hypertrophy also independent of high blood pressure through pathologic signaling mechanisms involving cardiac myocytes and immune cells (92). However, the role of angiotensin II in health and disease is multifactorial, cardiac myocytes and fibroblasts express angiotensin II-type 1 and 2 receptors (AT1R, AT2R). Additionally, as we recently demonstrated, isolated cardiac myocytes and fibroblasts of neonatal rats clearly express angiotensinogen (*Agt*), which was affected by FGF23 treatment *in vitro*, although *Agt* mRNA levels are frequently higher in cardiac myocytes than in fibroblasts (87). In addition, pro-fibrotic and pro-hypertrophic molecules, i.e., TGF-β, collagen 1, CTGF, and endothelin-1, known to be induced by AngII and/or aldosterone, are stimulated by FGF23 treatment in both cardiac myocytes and fibroblasts. Importantly, both AngII and aldosterone significantly up-regulate endogenous FGF23 expression in cardiac myocytes and induce hypertrophic cell growth, *βMHC, ANP*, and *BNP*. Whether these pathologies promoting cardiac hypertrophy and fibrosis are directly due to RAAS-mediated up-regulation of FGF23 in the heart or indirectly through the FGF23-mediated stimulation of RAAS is not clear. However, cardiac myocytes and fibroblasts may be potent target cells for local, non-canonical RAAS activation and paracrine FGF23 signaling (Figure 1). Taken these findings together, RAAS is an important regulator and key player in cardiovascular remodeling, resulting in HF that promotes sympathetic activation, systemic inflammation, and pathological cardiac hypertrophy. In turn, AngII and aldosterone induce FGF23 expression in cardiac myocytes in a feed-forward loop, although the underlying molecular mechanisms remain to be identified.

## Future Perspectives and Potential Therapeutic Options to Prevent FGF23-Asssociated Cardiovascular Pathologies

The possibilities of a therapeutic intervention to reduce or even prevent FGF23-associated cardiovascular diseases are certainly more diverse. Direct targeting of the FGF23/FGFR signaling complex is theoretically a straightforward option. It was shown that treatment with a monoclonal anti-FGF23 neutralizing antibody improved secondary hyperparathyroidism with increased vitamin D synthesis and normalization of bone markers in a rat model of experimental uremia (105). Nevertheless, chronic inhibition of FGF23 resulted in increased calcium and phosphate load, enhanced aortic calcification, and higher mortality of CKD rats compared to controls. In contrast, anti-FGF23 antibody therapy was proven to be effective and safe to cure bone abnormalities in hereditary causes of elevated FGF23 levels but reduced serum phosphate levels, e.g., X-linked hypophosphatemic rickets (106). Taken together, in states of high phosphate, general blocking of FGF23 is not suitable. Regarding the heart, the physiological relevance of locally produced FGF23 in the heart and the specific (side) effects of cardiac FGF23 suppression in health and disease are yet not clear and has to be elucidated in the future.

Targeting specific FGFRs may be another efficient strategy for therapeutic interventions with lesser side effects. An overview of FGFR small-molecule compounds, monoclonal antibodies, and FGFR analogs currently under development for the use as pro- or anti-FGF signaling therapeutics among others for the treatment of cardiovascular diseases in humans, is nicely summarized in a Review by Katoh (107). Regarding the treatment of LVH, Grabner and colleagues impressively demonstrated that targeting FGFR4 specifically prevents FGF23-induced cardiac hypertrophy *in vitro* and *in vivo* in CKD and non-CKD models (37). However, the specific FGFRs mediating the FGF23-induced signaling pathways in cardiac fibroblasts and other non-cardiac myocyte cells, such as endothelial cells and macrophages, is not finally discovered so fare. Moreover, whether blocking of FGFR4 also ameliorates cardiac fibrosis, improve endothelial function or reduce inflammatory processes is not known. In addition, the translation of this approach into clinical settings has to be investigated in future clinical studies.

The involvement of FGF23 in the RAAS activation is of particular interest and might be another promising target pathway for the therapeutic intervention of FGF23-mediated cardiac pathology. In patients with stable ischemic heart disease (SIHD) within the Prevention of Events With Angiotensin-Converting Enzyme (PEACE) trial, high FGF23 levels were independently associated with risk of HF or cardiovascular mortality (108). Furthermore, ACE inhibitor (ACEi) therapy reduced the incident of both events only in the patient subgroup with the highest FGF23 quartile. A clinical study in patients with advanced chronic systolic HF showed that the absence of ACEi therapy independently associated with increased circulating FGF23 levels (59). Interestingly, in this study, ACEi therapy was further associated with a lower risk of death, urgent heart transplantation, and ventricular assist device implantation only in HF patients without CKD in the highest FGF23 tertile, but not in those with CKD. The authors conclude that the association of FGF23 with adverse events reflects the activation of RAAS. In a recently published large multicentric European prospective observational study in patients with new onset and worsening HF (BIOSTAT-CHF trail), patients with the highest FGF23 quintile presented with worse renal function, more severe HF, increased congestion, and enhanced renin and aldosterone levels suggesting pronounced RAAS activation (109). Furthermore, higher plasma levels of FGF23 were independently associated with higher aldosterone levels and less successful uptitration of ACEi and ARB in addition to increased risk of mortality and HF hospitalization. In contrast, the use of beta-blocker was not significantly associated with high FGF23 concentrations. In CKD patients, it was shown that high FGF23 levels correlated with impaired ACE inhibition (110). In addition, CKD patients with the highest FGF23 responded with less effective ACEi therapy.

However, all of these studies hypothesized that increased FGF23 levels may provide additional information regarding the tolerability and effectiveness of RAAS blockade and may identify a subset of patients with SIHD or HF with or without CKD which may benefiting from an ACEi therapy. Nevertheless, only the effects of systemic RAAS inhibition on circulating FGF23 levels were investigated and very little is known about the impact of the local RAAS/FGF23 system in the heart. It is unknown if systemic treatment with RAAS inhibitors effectively target cardiac FGF23. A small cohort study in pediatric CKD patients on dialysis treatment or with functioning renal allograft failed to associate the expression of cardiac FGF23 with RAAS medications (25). One can speculate that the impaired efficiency of ACEi therapy in renal insufficiency is caused by additional overexpression of FGF23 in the heart, which may be resistant to systemic RAAS inhibition. This hypothesis is supported by the recently reported interactions of cardiac FGF23 synthesis and RAAS (87). AngII and aldosterone induce endogenous FGF23 in the heart and FGF23 stimulates *AGT* expression as well as the activation of RAAS in a feed forward loop, which may result in a resistance of therapeutic intervention.

Another important point is that most clinical trials determine the circulating levels of C-term FGF23. What is about the intact FGF23 protein in HF patients, which is discussed to be more important to mediate signaling events, and do RAAS inhibitory therapeutics impact on the posttranslational modification and/or cleavage of circulating and cardiac FGF23? It was shown in patients with autosomal dominant hypophosphatemic rickets, hyperphosphatemic familial tumoral calcinosis, and CKD, that the fraction of total FGF23 species representing intact biologically active hormone can markedly vary between different clinical settings (111). The cleavage of FGF23 in the bone is very well established, but less is known about the local processing of FGF23 in the heart and different cardiac cell types. The measurement of both biologically active and total FGF23 protein would represent an important advance for the understanding of its role also in different cardiovascular diseases. Moreover, future studies should provide more insights in the impact of therapeutic medications on the synthesis and cleavage of circulating and cardiac FGF23.

Important to note, treatment with ACEi or vitamin D was shown to improve soluble klotho levels in pediatric patients presenting with mild to moderate CKD (112). Taken together, these observations support the hypothesis of a direct cross talk between RAAS, vitamin D, FGF23, and klotho pathways, which can be targeted by RAAS inhibitor treatment. Normalization of soluble klotho may result in further modification of the specific FGFR for FGF23-mediated signaling in the heart though the interaction of FGF23 with klotho (65). It remains to be proven whether this will result in prevention of FGF23-driven pathological cardiac remodeling and counterregulate FGF23 cardiac toxicity.

## Summary

**Figure 2:**
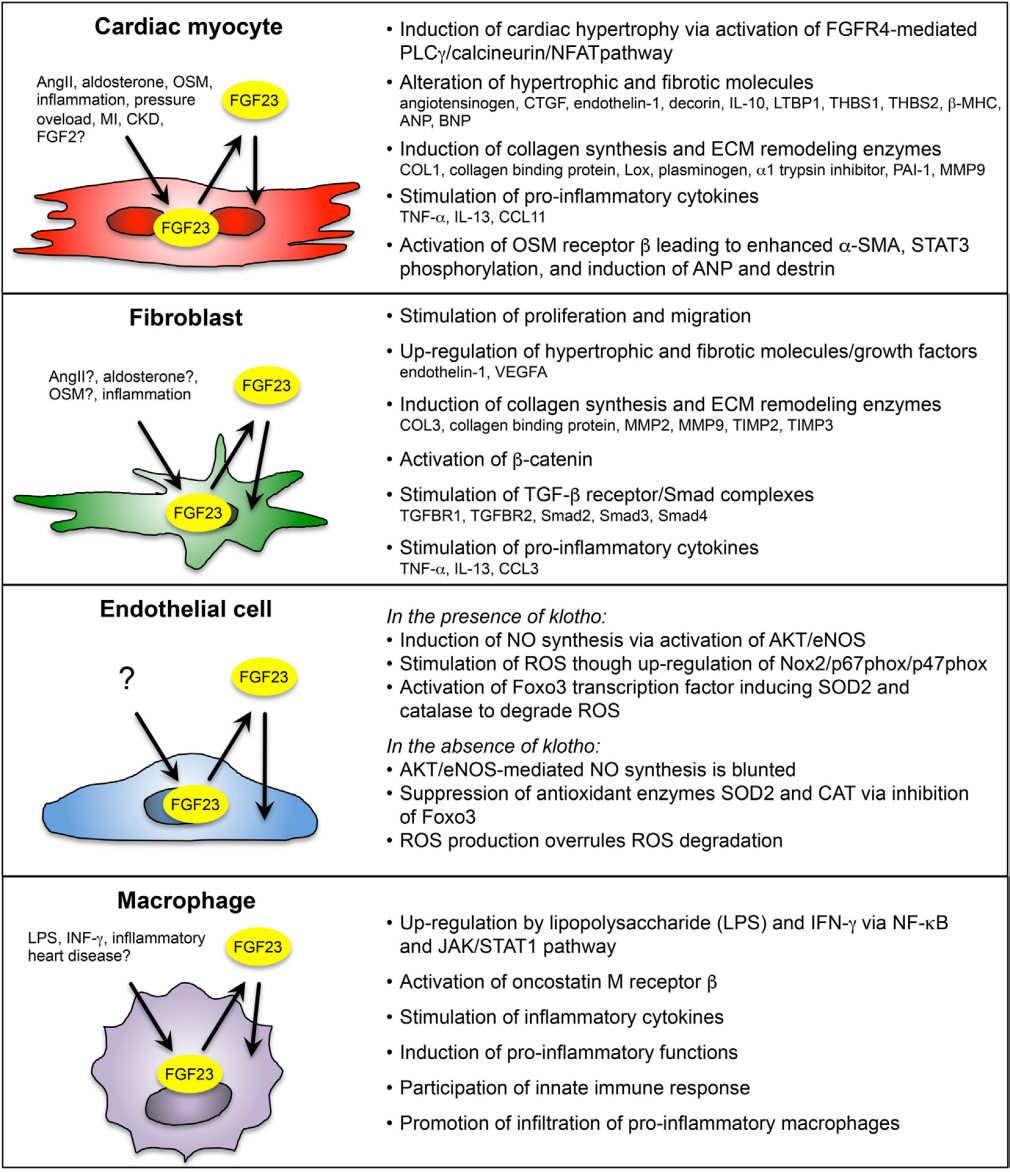
Overview of paracrine/autocrine acting FGF23 on cardiac myocyte and non-cardiac myocyte during cardiac remodeling and heart failure (HF). FGF23 is endogenously expressed in cardiac myocytes, fibroblasts, coronary artery endothelial cells, and macrophages in the heart. Depending on the cell type, FGF23 is induced by various pathogenic stimuli, including angiotensin II (AngII), aldosterone, oncostatin M (OSM), interferon γ (IFN- γ), lipopolysaccharide (LPS), inflammation, pressure overload, myocardial infarction (MI), and chronic kidney disease (CKD). Cardiac FGF23 exerts pro-hypertrophic, pro-fibrotic, and pro-inflammatory response in different cardiac cell types, which in turn activates pathologically phenotypic changes in the same or nearby cells through stimulation and secretion of growth factors, inflammatory cytokines, and pro-hypertrophic and pro-fibrotic molecules. [References for FGF23 in cardiac myocytes (23, 25, 26, 34, 37, 87, 102); cardiac fibroblasts (24, 87, 101); endothelial cells (90, 91); and macrophages (90, 95, 103).]

Enhanced cardiac FGF23 is reported in various clinical and experimental settings of cardiac remodeling or failure, e.g., CKD, ADHF, ischemic cardiomyopathy, myocarditis, dilated cardiomyopathy, inflammatory HF, TAC-induced pressure overload, experimental MI, and ischemia reperfusion in rodents. On a cellular level, FGF23 is expressed in cardiac myocytes and in other non-cardiac myocytes, including cardiac fibroblasts, vascular smooth muscle, and endothelial cells in coronary arteries, and in inflammatory macrophages (Figure 2).

**Figure 3:**
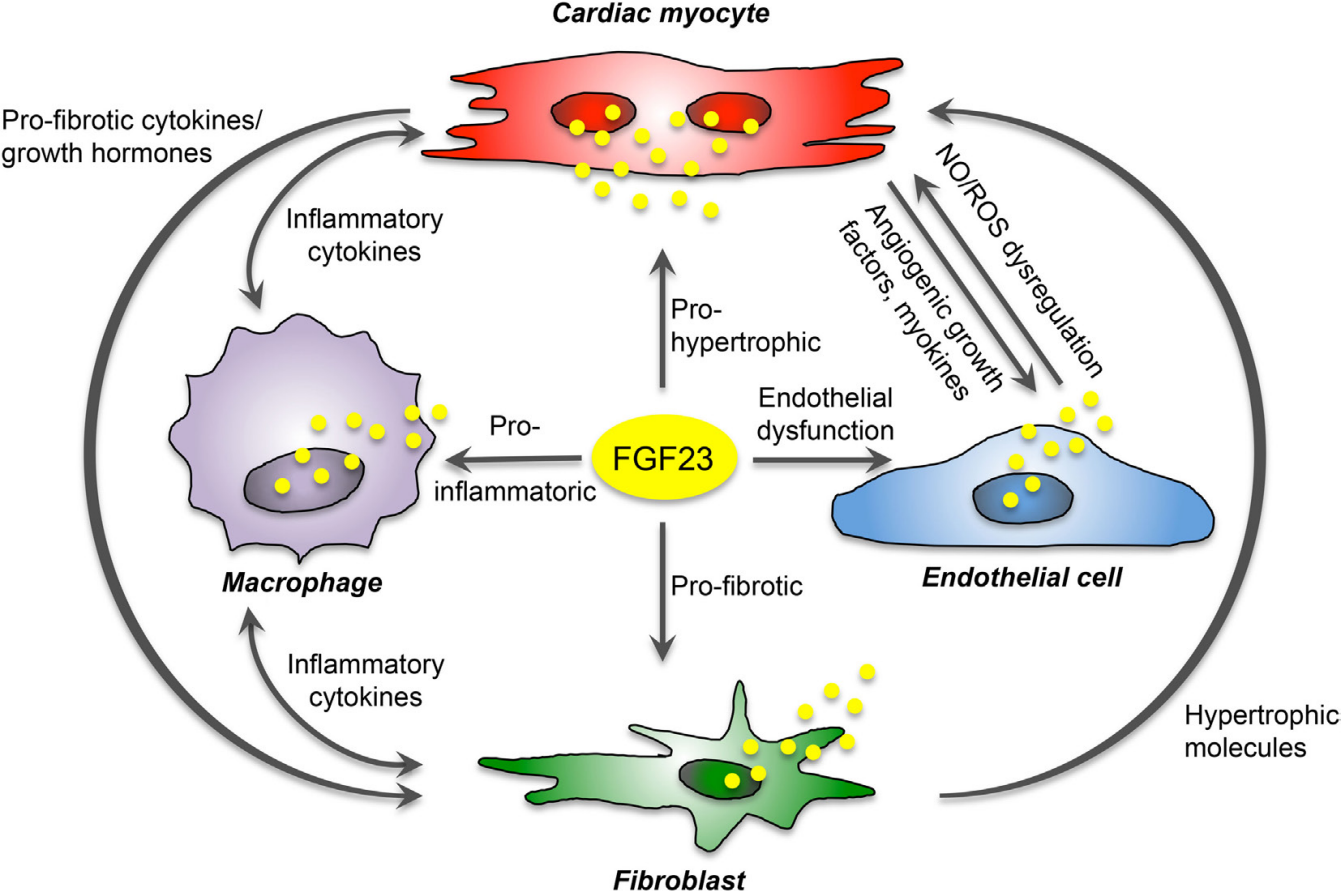
Proposed FGF23-mediated crosstalk between cardiac cells. FGF23 is expressed in cardiac myocytes, fibroblasts, endothelial cells, and macrophages, and directly induces pro-hypertrophic, pro-fibrotic, and pro-inflammatory signaling in a paracrine manner. Moreover, it promotes endothelial dysfunction in states of klotho deficiency. In addition, cardiac FGF23 stimulates different pathologic factors in each cardiac cell type and thereby participate indirectly in the crosstalk between cardiac myocytes and non-cardiac myocytes mediating cardiac hypertrophy and fibrosis.

The communication of cardiac myocytes and non-cardiac myocytes is mediated by intracellular signaling promoting pathologic ventricular remodeling and hypertrophy. Thereby, cardiac myocytes respond to stress stimuli with secretion of inflammatory cytokines, and damage-associated molecular patterns to impact on local non-cardiac myocytes. This activates resident macrophages and fibroblasts, which secrete pro-hypertrophic and pro-fibrotic cytokines to promote cardiac myocyte hypertrophy, fibroblast differentiation, matrix deposition, and finally interstitial myocardial fibrosis. Current data suggest that secreted by cardiac myocytes, FGF23 can stimulate pro-fibrotic factors in myocytes to induce fibrosis-related pathways in fibroblasts and consequently cardiac fibrosis in a paracrine manner, and while acting on cardiac myocytes, FGF23 directly induces pro-hypertrophic genes and promotes the progression of LVH in an autocrine fashion. In addition, *in vitro* studies suggest that FGF23 can induce endothelial dysfunction in the setting of klotho deficiency *via* enhanced ROS production and suppression of antioxidant enzymes resulting in increased oxidative stress, which in turn may contribute to the progression of cardiovascular disease in humans. Finally, endogenous FGF23 expression and secretion in infiltrating macrophages in the heart induce cardiovascular disease progression through induction of inflammation and fibrosis. Overall, despite the known expression in bone, FGF23 is now established as also synthesized and secreted in the heart of humans and rodents by different cardiac cell types. Therefore, cardiac FGF23 is involved in the complex interaction between cardiac myocytes, fibroblasts, endothelial cell, and macrophages to induce signaling pathways and phenotypic changes within the same or nearby cells in an autocrine or paracrine manner, respectively (Figure 3).

Nevertheless, recent studies suggest that FGF23 is involved in the activation of local RAAS and, in turn, AngII and aldosterone as active RAAS components induce the expression of FGF23 in the heart in a feed-forward loop. It is known that RAAS activation promotes pro-inflammatory, pro-fibrotic, and pro-hypertrophic processes in cardiac cells. Since AT1R is present in cardiac myocytes, fibroblasts, and macrophages, these cell types are potent target cells for local, non-canonical RAAS activation and paracrine FGF23 signaling.

However, hard evidence showing that enhanced cardiac FGF23 directly induces pathologic cardiac remodeling and hypertrophy in a paracrine manner is lacking. The induction of cardiac FGF23 may also be a consequence of the failing heart. Whether cardiac FGF23 alone is sufficient to induce spontaneous cardiac hypertrophy and fibrosis and whether high cardiac synthesis of FGF23 always leads to the progression of HF must be investigated in further studies. Finally, which factor directly stimulates cardiac FGF23 expression and under which conditions cardiac FGF23 leads to pathological changes of the heart must also be clarified in suitable experimental studies and in specific animal models.

## Author Contributions

ML-N and DH equally contributed to conception and design of the manuscript; ML-N performed extensive literature search and wrote the first draft of the review; DH critically reviewed the manuscript. Both authors read and approved the final manuscript.

## Conflict of Interest Statement

ML-N was supported by Amgen and received a research grant from the German Cardiac Society. DH received consultancy fees and/or research grants from Amgen, Chiesi Farmaceutici, Horizon Pharma, Kyowa Kirin, Pfizer, and Sandoz.
